# Safety and Efficacy of Liver-Directed Radiotherapy in Combination With Lenvatinib for Hepatocelluar Carcinoma With Macroscopic Tumor Thrombosis

**DOI:** 10.3389/fonc.2022.888755

**Published:** 2022-05-11

**Authors:** Jeong Il Yu, Wonseok Kang, Gyu Sang Yoo, Myung Ji Goh, Dong Hyun Sinn, Geum-Youn Gwak, Yong-Han Paik, Moon Seok Choi, Joon Hyeok Lee, Kwang Cheol Koh, Seung Woon Paik, Jung Yong Hong, Ho Yeong Lim, Boram Park, Hee Chul Park

**Affiliations:** ^1^ Samsung Medical Center, Sungkyunkwan University School of Medicine, Seoul, South Korea; ^2^ Department of Medicine, Samsung Medical Center, School of Medicine, Sungkyunkwan University, Seoul, South Korea; ^3^ Department of Health Sciences and Technology, Samsung Advanced Institute for Health Sciences and Technology (SAIHST), Sungkyunkwan University, Seoul, South Korea; ^4^ Biomedical Statistics Center, Research Institute for Future Medicine, Samsung Medical Center, Seoul, South Korea; ^5^ Department of Medical Device Management and Research, Samsung Advanced Institute for Health Sciences and Technology (SAIHST), Sungkyunkwan University, Seoul, South Korea

**Keywords:** hepatocellular carcinoma, radiotherapy, lenvatinib, efficacy, safety

## Abstract

**Background:**

This study aimed to compare the clinical outcomes of patients with hepatocellular carcinoma (HCC) and macroscopic tumor thrombosis who were treated with lenvatinib with or without combined liver-directed radiotherapy (LRT).

**Methods:**

From the institutional registry, we enrolled 82 patients diagnosed with HCC involving macroscopic tumor thrombosis and treated with lenvatinib monotherapy (non-LRT group, n = 54, 65.9%) or lenvatinib in combination with LRT (LRT group, n = 28, 34.1%). Patients were classified into the LRT group if LRT was performed within 8 weeks of lenvatinib initiation.

**Results:**

During the median follow-up period of 5.4 (range 1.4 to 17.5) months, there was no significant difference between the two groups in terms of overall adverse events. Although there was no statistical difference between the two groups in terms of overall response rate (32.1% vs. 20.4%, p = 0.15), a significantly higher treatment response was observed in the LRT group in terms of intrahepatic tumor response (67.9% vs. 20.4%, p < 0.001). In the LRT group, there was a slight difference in overall survival compared to the non-LRT group (64.1% in the LRT group vs. 37.7% in the non-LRT group at 12 months, hazard ratio [HR], 0.54; 95% confidence interval [CI] 0.28–1.03; p = .06), although it did not reach a statistically significant level. However, progression-free survival (PFS, 67.2% in the LRT group vs. 35.0% in the non-LRT group at 6 months, HR 0.47; 95% CI 0.27–0.82; p = 0.008) and intrahepatic progression-free survival (IHPFS, 74.3% in the LRT group vs. 43.3% in the non-LRT group at 6 months, HR 0.45; 95% CI 0.25–0.81; p = 0.008) were significantly superior in the LRT group. This result was also reproduced in the multivariate analysis adjusted for α-fetoprotein, another significant prognostic factor in this study, and the well-known prognostic factors, namely the presence of main portal vein tumor thrombosis and albumin-bilirubin grade.

**Conclusions:**

The combination of lenvatinib and LRT is relatively safe and effective in increasing the intrahepatic tumor response and improving PFS and IHPFS in patients with HCC and macroscopic tumor thrombosis.

## Introduction

Primary liver cancer, accounting for approximately 80% of hepatocellular carcinoma (HCC), is the second leading cause of cancer-related deaths worldwide, although it ranks as the seventh most commonly diagnosed cancer in terms of incidence according to Global Cancer Statistics 2020 (1). Despite considerable advancements in antiviral agents and imaging studies with the application of early screening, a significant proportion of patients are still diagnosed with unresectable disease (2). Furthermore, recurrence occurs in approximately 50% of cases within three years, even after curative treatment based on an early diagnosis. Nevertheless, there is no widely accepted effective adjuvant treatment yet (3, 4). Systemic therapy is recommended as the first-line treatment for HCC accompanied by extrahepatic metastasis and macroscopic tumor thrombosis, which may reduce the effect of transarterial chemoembolization (TACE) (5). The non-inferiority of lenvatinib compared with sorafenib, which had previously been the only effective first-line systemic agent for advanced HCC, was confirmed in a randomized phase III trial (6). Additionally, enhancement of its therapeutic effect in combination with immune checkpoint inhibitors has recently expanded its scope in various types of cancer management, including HCC (7).

Expectations and applications of radiotherapy, which is another treatment method that has proven efficacy in the treatment of various tumors, are increasing with advanced image guidance and precise radiation delivery techniques in the management of HCC. In particular, favorable clinical outcomes have been reported with liver-directed radiotherapy (LRT) for macroscopic tumor thrombosis, which is one of the main causes of intra- and extra-hepatic metastases and liver function deterioration (8–10). Although LRT can obtain effective local tumor control for tumor thrombosis and primary lesions, intrahepatic but outside of LRT and extrahepatic metastases and tumor progression are well-known patterns of treatment failure. For these reasons, the number of studies on the combination of LRT and systemic therapies is increasing in patients with HCC, especially those with macrovascular tumor thrombosis (11, 12).

Hence, we conducted this retrospective cohort study to compare the efficacy and safety of LRT in combination with lenvatinib for the treatment of HCC with macroscopic tumor thrombosis.

## Materials and Methods

This single-institutional retrospective cohort study was approved, with the requirement for informed consent waived because we used only de-identified, routinely collected data during hospital visits by the Samsung Medical Center Institutional Review Board.

The potential inclusion criteria of the present study were unresectable HCC diagnosed histologically and clinically according to the 2018 Korean Liver Cancer Association-National Cancer Center Korea practice guidelines and treated with lenvatinib at Samsung Medical Center from October 2018 to December 2020. Additionally, the subjects of the present study were selected according to the following criteria: 1. macroscopic tumor invading the portal vein, hepatic vein, and bile duct diagnosed using dynamic contrast-enhanced multi-phase computed tomography (CT), magnetic resonance (MR) imaging scans, and angiographic findings; 2. absence of previous upper abdominal radiotherapy; 3. pathologically confirmed tumors of other histological types compared to HCC; and 4. uncertain viable intrahepatic tumors.

During the study period, lenvatinib was recommended as the first-line systemic therapy for advanced HCC with widespread distant metastasis at diagnosis or for locally advanced HCC that failed or could not be treated locally. The initial dose and dose modification criteria were described in detail in a previous report (13).

LRT was recommended in cooperation with hepatologists, medical oncologists, and radiation oncologists for intrahepatic or extrahepatic lesions according to the 2018 Korean Liver Cancer Association-National Cancer Center (KLCA-NCC) guidelines (14). In particular, LRT mainly targets tumor thrombosis to suppress intrahepatic and extrahepatic metastasis and maintain liver function with or without intrahepatic primary tumor lesions. The primary intrahepatic tumor which caused the thrombosis was intended to be included in LRT targets. However, the target was limited to tumor thrombosis if the total liver volume receiving more than 30 Gy was 40% or higher (14).

Proton beam therapy (PBT) is preferentially considered for suitable cases when optimal respiratory control and avoidance of gastrointestinal radiation exposure are possible. In the other cases, photon radiotherapy (RT) was performed. The detailed protocol for photon radiotherapy and PBT was described in a previous study (15). In the present study, patients were classified into the LRT group if photon radiotherapy or PBT was performed within 8 weeks of lenvatinib initiation for HCC.

Treatment-related toxicities, including lenvatinib and LRT, were graded according to the Common Terminology Criteria for Adverse Events version 5.0 (CTCAE V5.0). Patients were evaluated in the second week of lenvatinib initiation and once a week during LRT using laboratory tests and physical findings. Assessment of treatment-related toxicities was performed every one to three months thereafter.

Treatment responses and disease progression were assessed 6 to 12 weeks after the initiation of lenvatinib with or without LRT by contrast-enhanced dynamic CT and MR images using the modified Response Evaluation Criteria in Solid Tumors (mRECIST) (10). Follow-up evaluation using contrast-enhanced dynamic CT and/or MR images was recommended every 1–3 months thereafter until disease progression was confirmed. The overall response rate, calculated as the sum of the complete response (CR) and partial response (PR), was defined as the best response recorded between the initiation of lenvatinib and disease progression. Additionally, intrahepatic progression was defined as an increase of 20% in the diameter of the viable tumor in the liver. In-field progression was defined as progression within the planning target volume defined as adding 5 mm to the clinical target volume of LRT, and otherwise, intrahepatic progression was defined as out-field progression.

To compare the differences between the groups, the chi-square test or Fisher*’*s exact test, and the Mann–Whitney U-test were used. Overall survival (OS), intrahepatic progression-free survival (IHPFS), and progression-free survival (PFS) were calculated as the duration from the starting date of primary treatment to the date when a new event was first detected or to the date of the last follow-up visit. Survival rates were estimated using the Kaplan–Meier method.

Multicollinearity was examined using the variance inflation factor (VIF), and a lack of multicollinearity was confirmed when the VIF was < 4. The univariable and multivariable Cox proportional hazards models were applied to the survival outcomes of PFS, IHPFS, and OS. Variables with a p-value < 0.2 in the univariable model were included in the multivariate model, and backward variable selection was performed, with an exclusion criterion of 0.05. Although the albumin-bilirubin (ALBI) and main portal vein tumor thrombosis (PVTT) were not statistically significant, these factors were included in the multivariate model due to their clinical significance. We additionally performed subgroup analysis to estimate the effect of LRT in each clinical subset. All statistical analyses were performed using SAS version 9.4 (SAS Institute, Cary, NC, USA) and R 3.3.2 (Vienna, Austria; http://www.R-project.org/). Statistical significance was set at p < 0.05, and all statistical tests were two-sided.

## Results

### Patients

**Figure 1:**
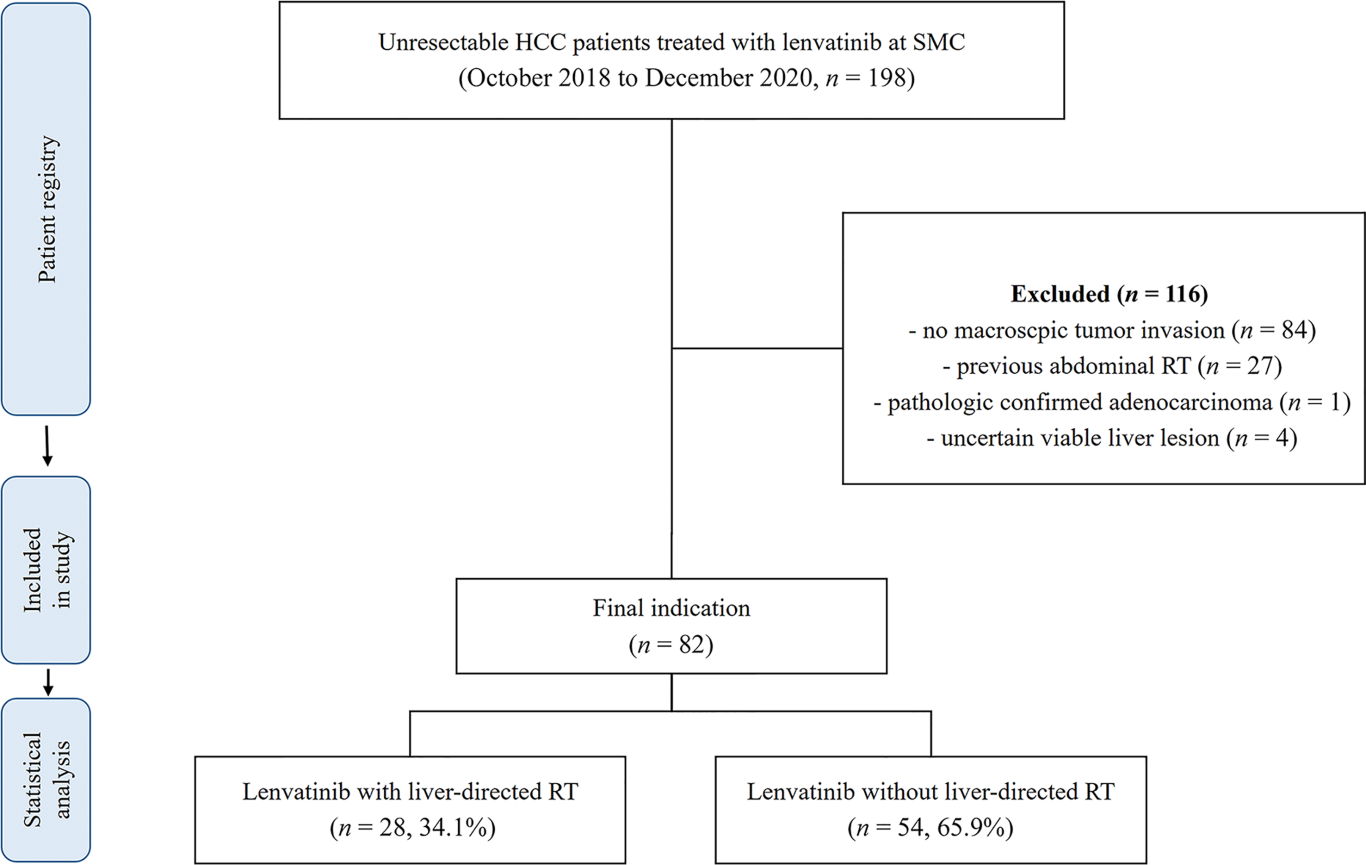
Flow chart of patient selection.

During the study period from October 2018 to December 2020, 198 unresectable HCC patients were treated with lenvatinib at the Samsung Medical Center. Among the 198 patients, 28 received LRT within 8 weeks of lenvatinib initiation out of 82 patients who met the inclusion criteria for the present study. A detailed flow diagram of patient selection for the present study is shown in **Figure 1**. LRT was started mostly within 1*–*2 weeks after the initiation of lenvatinib administration (range: eight weeks before to seven weeks after lenvatinib initiation), with six cases where the difference was more than four weeks. Additionally, in five cases, LRT was performed prior to lenvatinib initiation.

The baseline characteristics of the patients according to the combination of LRTs are displayed in **Table 1**. There were no significant differences in age, sex, etiology of HCC, Eastern Cooperative Oncology Group (ECOG) performance status, Child–Pugh score, ALBI score, TNM stage, or previous treatment between the two groups. PVTT of Vp3/4, which was defined according to a Japanese classification (16), was more frequent in the LRT group, and α-fetoprotein (AFP) and prothrombin-induced by vitamin K absence or antagonist-*II* (PIVKA-II) levels were lower in the LRT group than in the non-LRT group.

**Table 1:** Baseline characteristics of patients treated with or not treated with liver directed radiotherapy (LRT).

Characteristics	LRT group (n = 28)	Non-LRT group (n = 54)	p-value
Age (year)			
median range	56 39~77	57 35~80	0.52
Sex			
Male Female	22 (78.6) 6 (21.4)	46 (85.2) 8 (14.8)	0.54
Etiology			
Alcohol HBV HCV Other	3 (10.7) 22 (78.6) 2 (7.1) 1 (3.6)	11 (20.4) 41 (75.9) 2 (3.7) 0 (0.0)	0.33
ECOG performance status			
0 1	25 (89.3%) 3 (10.7%)	43 (79.6%) 11 (20.4%)	0.36
Child-Pugh score			
5 6 7 8	24 (85.7) 4 (14.3) 0 (0.0) 0 (0.0)	40 (74.1) 12 (22.2) 1 (1.9) 1 (1.9)	0.74
ALBI score median range	-2.77 -3.24 ~ -1.64	-2.70 -3.59 ~ -1.50	0.17
Vp3/4 PVTT Yes no	19 (67.9) 9 (32.1)	23 (42.6) 31 (57.4)	**0.04**
AFP (ng/mL)			**0.03**
median range	171 1.9-113429	827 1.5-1089000	
PIVKA-II (mAU/mL)			**0.004**
median range	576 8-42143	6072 29-300000	
T stage			
T2-3 T4	7 (25.0) 21 (75.0)	12 (22.2) 42 (77.8)	0.79
N stage			
N0 N1	18 (64.3) 10 (35.7)	36 (66.7) 18 (34.3)	1.00
M stage			0.82
M0 M1	16 (57.1) 12 (42.9)	28 (51.9) 26 (48.1)	
Previous treatment			
Surgical resection Radiofrequency ablation TARE TACE Radiotherapy Systemic therapy	5 (17.9) 2 (7.1) 1 (3.6) 7 (25.0) 8 (28.6) 0 (0.0)	7 (13.0) 3 (5.6) 1 (1.9) 10 (18.5) 4 (7.4) 1 (1.9)	0.74 1.00 1.00 0.57 **0.02** 1.00

Data are presented as median (range) or n (%). HBV, hepatitis B virus; HCV, hepatitis C virus; ECOG PS, Eastern Cooperative Oncology Group performance status; ALBL, albumin-bilirubin; PVTT, portal vein tumor thrombosis; AFP, alpha-fetoprotein; TARE, transarterial radio-embolization, TACE, transarterial chemo-embolization.Bold indicates statistical significance (p<0.05).

In the LRT group, PBT was used in 12 patients, while the remaining 16 patients were treated with photon radiotherapy. The median daily fraction size of LRT was 4.0 gray (Gy, range, 2.0 to 12.0 Gy), and the total dose was 35 Gy (range, 15.0 to 70 Gy). And, as a biological equivalent dose with α/β=10, the median total LRT dose was 47.0 Gy_10_ (range, 21.0 to 132.0 Gy_10_). In terms of LRT targets, 10 patients were irradiated for tumor thrombosis only, and the remaining 18 patients were treated for both tumor thrombosis and primary lesions causing tumor thrombosis.

### Treatment Response

In terms of overall response, CR was not observed, while PR was observed in 20 (24.4%) patients and stable disease (SD) in 36 (43.9%) based on the mRECIST criteria. PR was identified in 30 (37.7%) patients and SD in 30 (37.7%) when limited to the intrahepatic tumor response. Although there was no statistical difference between the two groups in terms of overall objective response rate (9 in 28 [32.1%] of LRT vs. 11 in 54 [20.4%] of non-LRT, p = 0.15), a significantly higher treatment response was observed in the LRT group in terms of intrahepatic tumor response (19 in 28 [67.9%] of LRT vs. 11 in 54 [20.4%] of non-LRT, p < 0.001). The tumor thrombosis, intrahepatic, and overall treatment responses of both groups are presented in **Supplementary Table 1**.

### Adverse Events According to Primary Treatment

**Table 2** shows the outcomes of treatment-related toxicities according to the combination of LRT (CTCAE V5.0). There were no significant differences between the two groups. In particular, grade 3 or 4 severe toxicities were rarely detected, with less than 5% in both groups. Only grade 1*–*2 anorexia was slightly higher in the LRT group than that in the non-LRT group (p = 0.05). In the LRT group, emergency surgery was required in one case four months after LRT completion due to duodenal perforation. The patient received 10 fractions of respiration gated intensity-modulated LRT of 3 Gy to PVTT only, and 29.2 Gy for 0.1 cc and 18.5 Gy for 3.0 cc of the duodenum.” Additionally, there was no significant difference in the incidence of gastroduodenal ulcers overall (2 in LRT vs. 3 in non-LRT, p = 0.35). Interruption/discontinuation or dose reduction of lenvatinib was performed in 40 (48.8%) patients, and there was no difference between the two groups (14 in LRT vs. 26 in non-LRT, p = 1.00).

**Table 2:** Adverse events of patients treated with or not treated with liver directed radiotherapy (LRT).

Toxicity		LRT group (n = 28)	Non-LRT group (n = 54)	p-value
Hand-foot skin reaction	grade 1/2 grade 3/4	4 (14.3) 1 (3.6)	15 (27.8) 0 (0.0)	0.16
Abdominal pain	grade 1/2 grade 3/4	10 (35.7) 1 (3.6)	14 (25.9) 0 (0.0)	0.19
Diarrhea	grade 1/2 grade 3/4	3 (10.7) 0 (0.0)	7 (13.0) 1 (1.9)	1.00
Hypertension	grade 1/2	8 (28.6)	10 (18.5)	0.40
Anorexia	grade 1/2	9 (32.1)	7 (13.0)	0.05
Nausea/vomiting	grade 1/2	5 (17.9)	5 (9.3)	0.30
Constipation	grade 1/2	4 (14.3)	5 (9.3)	0.48
Proteinuria	grade 1/2	2 (7.1)	4 (7.4)	1.00
Alopecia	grade 1/2	1 (3.6)	0 (0.0)	0.34
Hypothyroidism	grade 1/2	1 (3.6)	3 (5.6)	1.00
Hoarseness	grade 1/2	3 (10.7)	1 (1.9)	0.29
Skin rash	grade 1/2	2 (7.1)	3 (5.6)	1.00
Elevated AST/ALT	grade 1/2 grade 3/4	2 (7.1) 0 (0.0)	3 (5.6) 2 (3.7)	0.84
Headache	grade 1/2	1 (3.6)	4 (7.4)	0.66
Myalgia/Athalgia	grade 1/2	0 (0.0)	2 (3.7)	0.55
Oral mucositis	grade 1/2	2 (7.1)	3 (5.6)	1.00
Prutitus	grade 1/2	1 (3.6)	3 (5.6)	1.00
Varix bleeding	grade 1/2 grade 3/4	0 (0.0) 0 (0.0)	3 (5.6) 1 (1.9)	0.70
Epistaxis	grade 1/2	0 (0.0)	1 (1.9)	1.00
Gastroduodenal ulcer	grade 2 grade 4	1 (3.6) 1 (3.6)	3 (5.6) 0 (0.0)	0.35

Data are presented as n (%). AST, aspartate aminotransferase; ALT, alanine aminotransferase.

### Duration of Lenvatinib Treatment and Liver Function Maintenance

Although there was no statistical difference in the duration of lenvatinib use between the two groups, the LRT group showed a trend toward longer treatment (p = 0.07). The median duration of lenvatinib treatment in the non-LRT group was 3.9 (range, 1.5 to 22.9) months, while that in the LRT group was 7.1 (range, 1.4 to 19.0) months. There was no significant difference between the two groups in the comparison of the Child–Pugh score, which increased by two or more points compared to the baseline, or decline of liver function status to Child–Pugh class C.

The figures regarding the maintenance period of lenvatinib and the time to liver function deterioration between the two groups are presented in **Supplementary Figures 1**, **2**.

### Patterns of Progression and Next-Line Treatment

During the follow-up (median, 9.4 months; range, 1.7 to 21.5 months), progressive disease (PD) was confirmed in 62 (74.4%) patients (18 [64.3%] patients in the LRT group, and 44 [81.5%] patients in the non-LRT group, p = 0.09). Among them, extrahepatic PD was the main pattern of progression in five (4 in LRT and 1 in non-LRT) patients, and combined intra-and extrahepatic PD was seen in 21 (7 in LRT and 14 in non-LRT) patients. In the remaining 36 (7 in LRT and 29 in non-LRT) patients, intrahepatic PD was the main cause of initial progression. Intrahepatic PD with or without extrahepatic PD was more frequently identified in the non-LRT group than in the LRT group (43 in 54 [79.6%] of non-LRT vs. 14 in 28 [50.0%] of LRT, p = 0.05). Among the 14 patients in the LRT group who demonstrated intrahepatic PD, four had in-field, and ten had out-field intrahepatic PD.

Of the 62 patients with confirmed PD, next-line treatment was performed in 32 patients. Sorafenib was most commonly used in 25 patients (11 in LRT and 14 in non-LRT), of which two patients in the non-LRT group received LRT concurrently. Six patients (1 in LRT and 5 in non-LRT) received nivolumab, and two patients in the non-LRT group received LRT concurrently. The other patient in the non-LRT group received regorafenib treatment.

### Survival Outcomes According to Combination of LRT

The IHPFS, PFS, and OS for all enrolled 82 patients at 6-months were 54.0%, 46.4%, and 77.4%, respectively. The Kaplan–Meier estimated survival curves are shown in **Supplementary Figure 3**.

**Figure 2:**
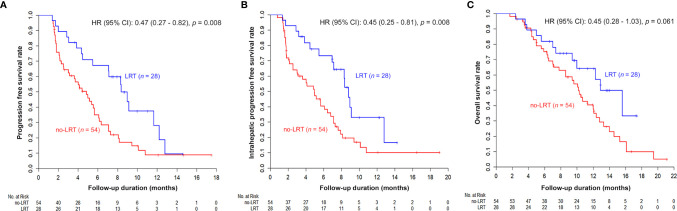
Kaplan–Meier estimated survival curves according to LRT: Although the difference of OS was marginal **(C)**, other survival curves were significantly better in the LRT group (PFS in **A** and IHPFS in **B**).

**Figure 3:**
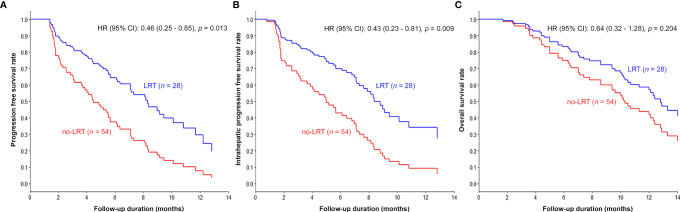
Kaplan–Meier curves adjusted for AFP, ALBI, and main PVTT according to LRT: The survival curves of LRT group were significantly prolonged compared to those of the non-LRT group (PFS in **A**, IHPFS in **B**, and OS in **C**).

The results of univariable and multivariable analyses of probable prognostic factors for survival outcomes are presented in **Table 3**. AFP (≥ 400 ng/ml) in all survival outcomes and T stage in OS were significant prognostic factors in both univariate and multivariate analyses. A significant difference between the two groups was found in IHPFS (p = 0.008, hazard ratio [HR] 0.45, 95% confidence interval [CI] 0.25–0.81) and PFS (p = 0.008, HR 0.47, 95% CI 0.27 – 0.82), but only a marginally significant difference in OS (p = 0.06, HR 0.54, 95% CI 0.28 – 1.03) was observed in the univariable analysis. In the multivariable analysis, significant difference in IHPFS (p = 0.009, HR 0.43, 95% CI 0.23 – 0.81) and PFS (p = 0.01, HR 0.46, 95% CI 0.25 – 0.85), and a non-significant difference in OS (p = 0.20, HR 0.64, 95% CI 0.32 – 1.28) were observed. The original and adjusted Kaplan–Meier estimated survival curves before and after adjusting for AFP, ALBI, and main PVTT are shown in **Figures 2**, **3**.

**Table 3:** Univariable analyses of probable prognostic factors on intrahepatic progression-free survival (IHPFS), progression-free survival (PFS) and overall survival (OS).

Variable		Total	PFS (N = 82, events = 62)					IHPFS (N = 82, events = 57)					OS (N = 82, events = 52)				
			Event	Univariable model		Multivariable model		Event	Univariable model		Multivariable model		Event	Univariable model		Multivariable model	
				HR (95% CI)	p	HR (95% CI)	p		HR (95% CI)	p	HR (95% CI)	p		HR (95% CI)	p	HR (95% CI)	p
Group	no-LRT	54	44	1 (ref)		1 (ref)		42	1 (ref)		1 (ref)		40	1 (ref)		1 (ref)	
	LRT	28	18	0.47 (0.27-0.82)	0.008	0.46 (0.25-0.85)	0.0127	15	0.45 (0.25-0.81)	0.0077	0.43 (0.23-0.81)	0.0089	12	0.54 (0.28-1.03)	0.061	0.64 (0.32-1.28)	0.2043
Age		82	62	0.98 (0.96-1.01)	0.177			57	0.99 (0.97-1.02)	0.5158			52	0.99 (0.97-1.02)	0.5692		
AFP (ng/ml)	<400	36	25	1 (ref)		1 (ref)		22	1 (ref)		1 (ref)		19	1 (ref)		1 (ref)	
	≥400	46	37	2.09 (1.22-3.58)	0.007	2.11 (1.21-3.68)	0.0087	35	2.07 (1.19-3.60)	0.0097	2.16 (1.21-3.84)	0.009	33	2.43 (1.35-4.38)	0.003	2.62 (1.41-4.86)	0.0024
PIVKA-II (mAU/ml)	<1000	33	28	1 (ref)				24	1 (ref)				19	1 (ref)			
	≥1000	48	34	1.13 (0.68-1.87)	0.64			33	1.32 (0.78-2.24)	0.3079			33	1.42 (0.81-2.52)	0.2248		
Sex	1	68	50	1 (ref)				46	1 (ref)				45	1 (ref)			
	2	14	12	1.46 (0.77-2.75)	0.245			11	1.56 (0.80-3.04)	0.1893			7	1.06 (0.48-2.36)	0.8807		
ECOG PS	0	68	53	1 (ref)				48	1 (ref)				41	1 (ref)			
	1	14	9	1.00 (0.49-2.03)	0.999				1.15 (0.56-2.35)	0.7007			11	1.28 (0.65-2.50)	0.4736		
Etiology	Others	15	10	1 (ref)				10	1 (ref)				11	1 (ref)			
	HBV	67	52	1.04 (0.53-2.04)	0.917			47	0.93 (0.47-1.84)	0.8351			41	0.67 (0.34-1.32)	0.2486		
Child-Pugh class	A	80	60	1 (ref)				55	1 (ref)				51	1 (ref)			
	B	2	2	1.18 (0.29-4.85)	0.821			2	1.33 (0.32-5.49)	0.6935			1	1.25 (0.17-9.24)	0.8239		
ALBI grade	1	48	37	1 (ref)		1 (ref)		34	1 (ref)		1 (ref)		32	1 (ref)		1 (ref)	
	2	34	25	1.00 (0.60-1.67)	0.996	0.68 (0.39-1.17)	0.1633	23	0.94 (0.55-1.59)	0.8035	0.62 (0.35-1.09)	0.0986	20	1.09 (0.62-1.91)	0.7741	1.13 (0.62-2.06)	0.6865
T stage	2-3	19	14	1 (ref)				11	1 (ref)				15	1 (ref)		1 (ref)	
	4	63	48	0.93 (0.51-1.69)	0.813			46	1.14 (0.59-2.20)	0.702			37	0.54 (0.29-1.00)	0.0499	0.39 (0.2-0.79)	0.0091
N stage	0	54	42	1 (ref)				39	1 (ref)				35	1 (ref)			
	1	28	20	0.69 (0.40-1.18)	0.175			18	0.67 (0.38-1.19)	0.17			17	0.62 (0.34-1.12)	0.1125		
M stage	0	44	32	1 (ref)				30	1 (ref)				26	1 (ref)			
	1	38	30	1.20 (0.73-1.97)	0.486			27	1.08 (0.64-1.82)	0.7743			26	1.49 (0.86-2.59)	0.1566		
main PVTT	0	40	31	1 (ref)		1 (ref)		28	1 (ref)		1 (ref)		27	1 (ref)		1 (ref)	
	1	42	31	0.81 (0.49-1.33)	0.401	0.98 (0.58-1.65)	0.9306	29	0.82 (0.49-1.38)	0.457	1.02 (0.59-1.76)	0.9357	25	0.83 (0.48-1.44)	0.5134	1.45 (0.76-2.76)	0.2595

PFS, progression-free survival; IHPFS, intrahepatic progression-free survival; OS, overall survival; HR, hazard ratio; CI, confidence interval; LRT, liver-directed radiotherapy; ECOG PS, Eastern Cooperative Oncology Group performance status; HBV, hepatitis B virus; ALBL, albumin-bilirubin; PVTT, portal vein tumor thrombosis.

We additionally performed subgroup analysis to estimate the effect of LRT in each clinical subset (**Supplementary Table 2**). In this analysis, the positive effect of LRT on survival outcomes showed a more pronounced difference in the subgroup associated with hepatitis B virus (HBV) and ALBI grade 1.

In the LRT group, there was no significant difference in terms of IHPFS, PFS, and OS when comparing PBT to photon radiotherapy. Additionally, there was no difference in LRT targets; however, OS was found to be significantly superior in patients in which both tumor thrombosis and the primary lesions were treated. The Kaplan–Meier estimated survival curves according to the method of radiotherapy and LRT targets are presented in **Supplementary Figures 4**, **5**.

## Discussion

In the present study, we evaluated the efficacy and safety of LRT in combination with lenvatinib for treating advanced HCC with macroscopic tumor thrombosis. The oncologic outcomes from the LRT combination were generally superior to lenvatinib alone, with comparable and acceptable treatment-related toxicities and maintenance duration of liver function and lenvatinib.

Systemic management for HCC, which has been stagnant for about 10 years since the effectiveness of sorafenib was confirmed in advanced HCC in 2007, is making rapid progress (17). The most important improvement in this aspect so far is the introduction of atezolizumab, which is one of the most popular immune checkpoint inhibitors (ICIs) and monoclonal antibodies against the programmed cell death-ligand 1. In combination with bevacizumab, it is superior in terms of survival outcomes and tumor response over the existing standard treatment, sorafenib, as the first-line agent through an international multicenter phase III randomized trial, the IMbrave150 study (18). Based on the positive outcomes of the IMbrave150 study, various studies evaluating the efficacy of combination treatment with ICIs for HCC are ongoing (7). Another important progress in the management of advanced HCC is the introduction of lenvatinib, a selective inhibitor of tyrosine kinase involved in tumor angiogenesis and malignant transformation. In a multicenter, phase III randomized trial (REFLECT), lenvatinib demonstrated non-inferiority in terms of OS compared with sorafenib, which is the existing first-line standard of care for advanced HCC (6).

In the REFLECT study, lenvatinib proved its higher antitumor efficacy by showing a significantly higher objective response rate of more than triple that of sorafenib as 40.6% per investigator assessment using mRECIST. In addition, PFS and time to progression were significantly superior in the lenvatinib arm. Furthermore, the superiority of lenvatinib over sorafenib in terms of OS was observed when AFP was adjusted, although this was the result of a *post-hoc* analysis (19). Currently, clinical studies are ongoing to improve the treatment outcome in HCC through combination therapy, aiming for a high response rate of lenvatinib and a long-term maintaining effect of ICI (20).

Macroscopic tumor thrombosis in HCC is one of the most serious conditions associated with poor prognosis, representing the Barcelona Clinic Liver Cancer stage C (5). This condition is generally considered an indication for systemic agents rather than local therapies because of its association with rapid intrahepatic and extrahepatic metastases, as well as concerns about maintaining liver function (21). In certain patients with this condition, favorable long-term outcomes through surgical resection, including tumor thrombus, have been reported (10, 22). A recent report of a survey study from the Liver Cancer Study Group of Japan presented cumulative survival rates of 33.0% in Vp3 and 18.3% in Vp4 at 5 years after hepatic resection (23). In our previous study, we confirmed that obtaining an early treatment response to macroscopic portal vein tumor thrombosis by TACE and RT was clearly associated with reduced intrahepatic and extrahepatic metastases and preserved liver function status (24). In addition, a phase III randomized trial confirmed that in HCC with macroscopic vascular invasion, PFS and OS increased through TACE plus RT and was more effective than sorafenib treatment (9). Based on these results, there is a growing expectation for combination management with reliable local modalities and the introduction of effective systemic agents in these patients (25).

As one of the major locoregional modalities in oncologic fields, RT is used in more than 50% of cancer patients during their management for curative or palliative aims, and its usage is continuously increasing with technical advancement (26). In particular, research and application for the management of HCC, which clinicians have been hesitant to use owing to concerns about radiation-induced liver function deterioration, is rapidly increasing (27). Recently, a randomized phase III trial comparing the clinical outcomes of proton beam RT and radiofrequency ablation (RFA), which is one of the proven standard local modalities for recurrent or residual small HCC, demonstrated non-inferior outcomes of proton beam RT compared to RFA in terms of survival as well as local control (28). In another prospective, randomized, phase II trial to compare the efficacy and safety of TACE plus RT and sorafenib in HCC with macroscopic vascular invasion, TACE plus RT showed a higher PFS, OS, and objective response rate (more than six times than sorafenib by approximately 30%) than the standard treatment, which is sorafenib (9). Although the time to progression was much longer in the TACE plus RT group, intrahepatic and extrahepatic progression continued to occur in most patients, even in this group.

As mentioned above, considering the importance of obtaining an early treatment response through a reliable local modality and a widespread intrahepatic and extrahepatic progression pattern, the necessity of combination treatment with LRT and systemic agents is emphasized (29). Several studies have evaluated the efficacy and safety of combination treatment with RT and sorafenib in advanced HCC, and one meta-analysis showed that concurrent RT and sorafenib treatment was more beneficial than non-concurrent treatment (odds ratio 3.3, p = 0.015) (12). The superiority of RT combined with sorafenib over sorafenib alone (adjusted HR 0.57, 95% CI 0.51–0.63) has also been reported in other nationwide cancer registry-based studies from Taiwan (11). However, there are very limited reports on the use of a combination strategy of RT and lenvatinib or ICI, whose use is increasing due to proven clinical benefits in advanced HCC (30).

In the present study, based on the above background, the LRT combination did not show any increase in adverse events compared to lenvatinib alone. Furthermore, LRT clearly enhanced the treatment response in target lesions, which ultimately improved PFS by increasing IHPFS. It was also suggested that the duration of lenvatinib treatment could be extended. Based on our research results, we expect enhancement of clinical outcomes in HCC with macroscopic tumor thrombosis through a combination of LRT and lenvatinib by complementing each other’s limitations.

This study had several limitations. Above all, since it was designed as a retrospective real-world study, it is not free from selection bias between the two groups. Although we adjusted for possible confounders for a more accurate analysis between the two groups, it was impossible to control for all confounding variables. For example, differences in the severity and extent of extrahepatic metastasis might be present, even though we found no difference between the two groups regarding proportion of extrahepatic metastasis itself. Second, the sample size and duration of follow-up in the present study were limited and prevented drawing concrete conclusions. Third, the LRT methods, total doses, and targets varied greatly. Finally, more than three-quarters of enrolled patients had HBV-related HCC because the present study was conducted in Korea, which is an HBV-endemic region.

Despite these limitations, the present study was the only one thus far that compares clinical outcomes of LRT in combination with lenvatinib, and offers important and valuable findings of its efficacy and safety. Nevertheless, further large-scale studies are needed to verify the outcomes of this study.

## Conclusion

Combination treatment of lenvatinib and LRT for advanced HCC with macroscopic tumor thrombosis was found to be relatively safe and did not lead to a deterioration of liver function or a shortened duration of lenvatinib administration period. In terms of survival and intrahepatic tumor response, the oncologic outcomes of combination treatment are generally superior to that of lenvatinib alone.

## Data Availability Statement

All data generated or analyzed during this study are included in this article and/or its online **Supplementary Material** files. Further inquiries can be directed to the corresponding author.

## Ethics Statement

This study was performed in accordance with the Declaration of Helsinki, and it has been approved by the Institutional Review Board at the Samsung Medical Center (IRB No. 2021-06-204-001). As the study used only deidentified data routinely collected during hospital visits, the requirement of obtaining informed consent from the patients was waived.

## Author Contributions

JIY, HCP and WK: study design, statistical analysis, data interpretation, and drafting of the manuscript. GSY, MJG, DHS, GYG, YHP, JYH, BP, and MSC: data interpretation, study supervision, and critical revision of the manuscript JHL, KCK, SWP, and HYL: study supervision, and critical revision of the manuscript All authors approved the final draft of the manuscript.

## Funding

This research was supported by a grant of the Korea Health Technology R&D Project through the Korea Health Industry Development Institute (KHIDI), funded by the Ministry of Health & Welfare, Republic of Korea (HR20C0025). This research was also supported by the National Research Foundation of Korea (NRF) grant funded by the Korea government (MSIT) (NRF-2020R1F1A1073205, JY; NRF-2019R1C1C1007729, WK) and Future Medicine 20*30 Project of the Samsung Medical Center [SMX1220101 and SMO1220111].

## Conflict of Interest

The authors declare that the research was conducted in the absence of any commercial or financial relationships that could be construed as a potential conflict of interest.

## Publisher’s Note

All claims expressed in this article are solely those of the authors and do not necessarily represent those of their affiliated organizations, or those of the publisher, the editors and the reviewers. Any product that may be evaluated in this article, or claim that may be made by its manufacturer, is not guaranteed or endorsed by the publisher.
