# Supplementary material for: Safety and Efficacy of Liver-Directed Radiotherapy in Combination With Lenvatinib for Hepatocelluar Carcinoma With Macroscopic Tumor Thrombosis
Source: Front Oncol. 2022 May 11;12:888755. doi: 10.3389/fonc.2022.888755 (PMC9130955; doi:10.3389/fonc.2022.888755)

**Supplementary Table 1|** Treatment response evaluated based on the mRECIST criteria

|  | LRT group (n=28) | | | | Non-LRT group (n=54) | | | |  |
| --- | --- | --- | --- | --- | --- | --- | --- | --- | --- |
| Response | CR | PR | SD | PD | CR | PR | SD | PD | p |
| Tumor thrombosis | 1 (3.6) | 22 (78.6) | 5 (17.9) | 0 (0.0) | 0 | 15 (27.8) | 25 (46.3) | 14 (25.9) | <0.001 |
| Intrahepatic | 0 | 19 (67.9) | 6 (21.4) | 3 (10.7) | 0 | 11 (20.4) | 24 (44.4) | 19 (35.2) | <0.001 |
| Overall | 0 | 9 (32.1) | 14 (50.0) | 5 (17.9) | 0 | 11 (20.4) | 22 (40.7) | 21 (38.9) | 0.15 |

Data are presented as *n* (%). CR, complete response PR, partial response; SD, stable disease; PD, partial response

**Supplementary Table 2|** Univariable Cox proportional hazard model in subgroups.

| **Variable** |  | **Total** | **PFS (N = 82, events = 62)** | | | | | | **IHPFS (N = 82, events = 57)** | | | | | | **OS (N = 82, events = 52)** | | | | | |
| --- | --- | --- | --- | --- | --- | --- | --- | --- | --- | --- | --- | --- | --- | --- | --- | --- | --- | --- | --- | --- |
|  |  |  | **Non-LRT** | | **LRT** | | **reference (non-LRT)** | | **Non-LRT** | | **LRT** | | **reference (non-LRT)** | | **Non-LRT** | | **LRT** | | **reference (non-LRT)** | |
|  |  |  | **N** | **Event (%)** | **N** | **Event (%)** | **HR (95% CI)** | **p-value** | **N** | **Event (%)** | **N** | **Event (%)** | **HR (95% CI)** | **p-value** | **N** | **Event (%)** | **N** | **Event (%)** | **HR (95% CI)** | **p-value** |
| Age | ≤55 | 36 | 24 | 22 (91.7) | 12 | 8 (66.7) | 0.46 (0.20-1.04) | 0.062 | 24 | 21 (87.5) | 12 | 6 (50.0) | 0.39 (0.16-0.97) | 0.0435 | 24 | 18 (75.0) | 12 | 6 (50.0) | 0.99 (0.39-2.52) | 0.98 |
|  | >55 | 46 | 30 | 22 (73.3) | 16 | 10 (62.5) | 0.49 (0.23-1.04) | 0.064 | 30 | 21 (70.0) | 16 | 9 (56.3) | 0.51 (0.23-1.13) | 0.0954 | 30 | 22 (73.3) | 16 | 6 (37.5) | 0.33 (0.14-0.83) | 0.0183 |
| AFP (ng/ml) | <400 | 36 | 21 | 16 (76.2) | 15 | 9 (60.0) | 0.50 (0.22-1.13) | 0.0967 | 21 | 15 (71.4) | 15 | 7 (46.7) | 0.48 (0.19-1.18) | 0.109 | 21 | 15 (71.4) | 15 | 4 (26.7) | 0.34 (0.11-1.04) | 0.058 |
|  | ≥400 | 46 | 33 | 28 (84.9) | 13 | 9 (69.2) | 0.53 (0.25-1.14) | 0.1051 | 33 | 27 (81.8) | 13 | 8 (61.5) | 0.51 (0.23-1.14) | 0.1014 | 33 | 25 (75.8) | 13 | 8 (61.5) | 1.08 (0.48-2.44) | 0.8568 |
| PIVKA-II (mAU/ml) | <1000 | 33 | 17 | 16 (94.1) | 16 | 12 (75.0) | 0.43 (0.19-0.97) | 0.0427 | 17 | 15 (88.2) | 16 | 9 (56.3) | 0.41 (0.17-0.98) | 0.0447 | 17 | 12 (70.6) | 16 | 7 (43.8) | 0.56 (0.22-1.43) | 0.223 |
|  | ≥1000 | 48 | 36 | 28 (77.8) | 12 | 6 (50.0) | 0.36 (0.15-0.88) | 0.0257 | 36 | 27 (75.0) | 12 | 6 (50.0) | 0.42 (0.17-1.03) | 0.0579 | 36 | 28 (77.8) | 12 | 5 (41.7) | 0.50 (0.19-1.32) | 0.1613 |
| Sex | 1 | 68 | 46 | 37 (80.4) | 22 | 13 (59.1) | 0.44 (0.23-0.83) | 0.0114 | 46 | 36 (78.3) | 22 | 10 (45.5) | 0.36 (0.18-0.72) | 0.0043 | 46 | 35 (76.1) | 22 | 10 (45.5) | 0.60 (0.29-1.21) | 0.1542 |
|  | 2 | 14 | 8 | 7 (87.5) | 6 | 5 (83.3) | 0.37 (0.09-1.47) | 0.1587 | 8 | 6 (75.0) | 6 | 5 (83.3) | 0.54 (0.13-2.17) | 0.3836 | 8 | 5 (62.5) | 6 | 2 (33.3) | 0.38 (0.07-1.99) | 0.2523 |
| ECOG | 0 | 68 | 43 | 37 (86.1) | 25 | 16 (64.0) | 0.42 (0.23-0.76) | 0.0043 | 43 | 35 (81.4) | 25 | 13 (52.0) | 0.40 (0.21-0.77) | 0.0055 | 43 | 31 (72.1) | 25 | 10 (40.0) | 0.50 (0.24-1.02) | 0.0575 |
|  | 1 | 14 | 11 | 7 (63.6) | 3 | 2 (66.7) | 0.72 (0.14-3.58) | 0.6842 | 11 | 7 (63.6) | 3 | 2 (66.7) | 0.72 (0.14-3.62) | 0.6928 | 11 | 9 (81.8) | 3 | 2 (66.7) | 1.04 (0.21-5.04) | 0.9628 |
| Etiology | Others | 15 | 11 | 8 (72.7) | 4 | 2 (50.0) | 0.60 (0.13-2.87) | 0.5253 | 11 | 8 (72.7) | 4 | 2 (50.0) | 0.62 (0.13-2.94) | 0.5449 | 11 | 8 (72.7) | 4 | 3 (75.0) | 1.05 (0.27-4.09) | 0.9396 |
|  | HBV | 67 | 43 | 36 (83.7) | 24 | 16 (66.7) | 0.41 (0.23-0.76) | 0.0041 | 43 | 34 (79.1) | 24 | 13 (54.2) | 0.40 (0.21-0.77) | 0.0058 | 43 | 32 (74.4) | 24 | 9 (37.5) | 0.47 (0.22-0.99) | 0.0465 |
| Child-Pugh class | A | 80 | 52 | 42 (80.8) | 28 | 18 (64.3) | 0.47 (0.27-0.83) | 0.0088 | 52 | 40 (76.9) | 28 | 15 (53.6) | 0.45 (0.25-0.82) | 0.0089 | 52 | 39 (75.0) | 28 | 12 (42.9) | 0.54 (0.28-1.03) | 0.0629 |
|  | B | 2 | 2 | 2 (100.0) | 0 | (0) |  |  | 2 | 2 (100.0) | 0 | (0) |  |  | 2 | 1 (50.0) | 0 | (0) |  |  |
| ALBI | 1 | 48 | 29 | 25 (86.2) | 19 | 12 (63.2) | 0.25 (0.12-0.56) | 0.0007 | 29 | 24 (82.8) | 19 | 10 (52.6) | 0.29 (0.13-0.63) | 0.0019 | 29 | 25 (86.2) | 19 | 7 (36.8) | 0.37 (0.16-0.86) | 0.0206 |
|  | 2 | 34 | 25 | 19 (76.0) | 9 | 6 (66.7) | 0.71 (0.28-1.79) | 0.4676 | 25 | 18 (72.0) | 9 | 5 (55.6) | 0.67 (0.25-1.80) | 0.4222 | 25 | 15 (60.) | 9 | 5 (55.6) | 1.00 (0.35-2.85) | 0.9989 |
| T stage | 2-3 | 19 | 12 | 9 (75.0) | 7 | 5 (71.4) | 0.52 (0.16-1.70) | 0.2769 | 12 | 8 (66.7) | 7 | 3 (42.9) | 0.52 (0.14-2.02) | 0.3451 | 12 | 12 (100.0) | 7 | 3 (42.9) | 0.56 (0.16-2.03) | 0.379 |
|  | 4 | 63 | 42 | 35 (83.3) | 21 | 13 (61.9) | 0.44 (0.23-0.84) | 0.0122 | 42 | 34 (81.0) | 21 | 12 (57.1) | 0.43 (0.22-0.83) | 0.0115 | 42 | 28 (66.7) | 21 | 9 (42.9) | 0.57 (0.27-1.22) | 0.1491 |
| N stage | 0 | 54 | 36 | 31 (86.1) | 18 | 11 (61.1) | 0.32 (0.15-0.67) | 0.0029 | 36 | 29 (80.6) | 18 | 10 (55.6) | 0.34 (0.15-0.74) | 0.0064 | 36 | 26 (72.2) | 18 | 9 (50.0) | 0.52 (0.24-1.13) | 0.0981 |
|  | 1 | 28 | 18 | 13 (72.2) | 10 | 7 (70.0) | 0.71 (0.28-1.79) | 0.462 | 18 | 13 (72.2) | 10 | 5 (50.0) | 0.58 (0.20-1.66) | 0.3107 | 18 | 14 (77.8) | 10 | 3 (30.0) | 0.50 (0.14-1.78) | 0.2865 |
| M stage | 0 | 44 | 28 | 23 (82.1) | 16 | 9 (56.3) | 0.38 (0.17-0.84) | 0.0161 | 28 | 23 (82.1) | 16 | 7 (43.8) | 0.35 (0.15-0.82) | 0.0154 | 28 | 19 (67.9) | 16 | 7 (43.8) | 0.70 (0.29-1.68) | 0.4221 |
|  | 1 | 38 | 26 | 21 (80.8) | 12 | 9 (75.0) | 0.54 (0.24-1.20) | 0.1325 | 26 | 19 (73.1) | 12 | 8 (66.7) | 0.55 (0.24-1.27) | 0.1635 | 26 | 21 (80.8) | 12 | 5 (41.7) | 0.41 (0.15-1.10) | 0.0755 |
| main PVTT | 0 | 40 | 31 | 25 (80.7) | 9 | 6 (66.7) | 0.43 (0.17-1.07) | 0.0694 | 31 | 23 (74.2) | 9 | 5 (55.6) | 0.44 (0.17-1.18) | 0.1034 | 31 | 24 (77.4) | 9 | 3 (33.3) | 0.50 (0.15-1.68) | 0.2612 |
|  | 1 | 42 | 23 | 19 (82.6) | 19 | 12 (63.2) | 0.52 (0.25-1.07) | 0.0762 | 23 | 19 (82.6) | 19 | 10 (52.6) | 0.45 (0.21-0.97) | 0.041 | 23 | 16 (69.6) | 19 | 9 (47.4) | 0.58 (0.26-1.33) | 0.1992 |

*PFS, progression-free survival; IHPFS, intrahepatic progression-free survival; OS, overall survival; HR, hazard ratio; CI, confidence interval; LRT, liver-directed radiotherapy; ECOG PS, Eastern Cooperative Oncology Group performance status; HBV, hepatitis B virus; ALBL, albumin-bilirubin; PVTT, portal vein tumor thrombosis*

**Supplementary Figure 1 |** Maintenance period of lenvatinib between the LRT and non-LRT groups.


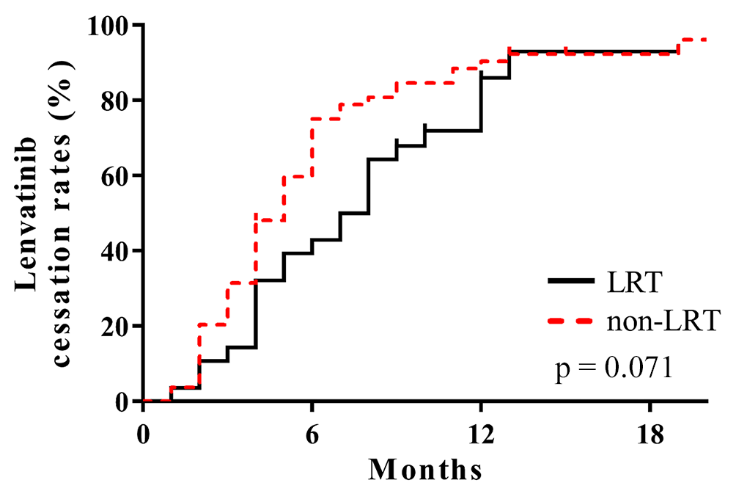


**Supplementary Figure 2 |** Time to liver function deterioration between LRT and non-LRT groups.


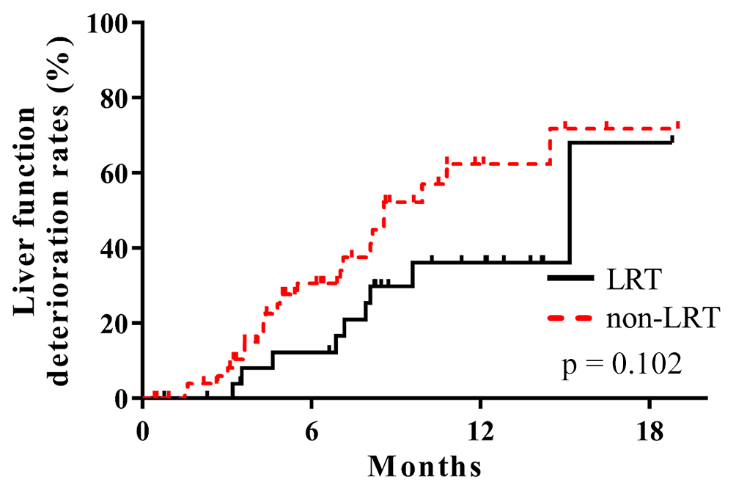


**Supplementary Figure 3 |** Kaplan–Meier estimated survival curves of intrahepatic-progression-free survival (IHPFS), progression-free survival (PFS), and overall survival (OS) for all enrolled 82 patients.


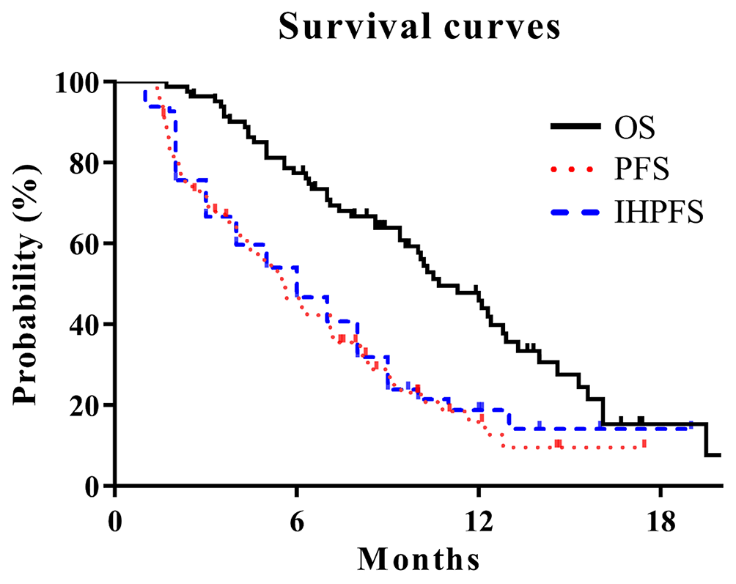


**Supplementary Figure 4 |** Kaplan–Meier estimated survival curves of intrahepatic-progression-free survival (IHPFS), progression-free survival (PFS), and overall survival (OS) according to the methods of LRT.


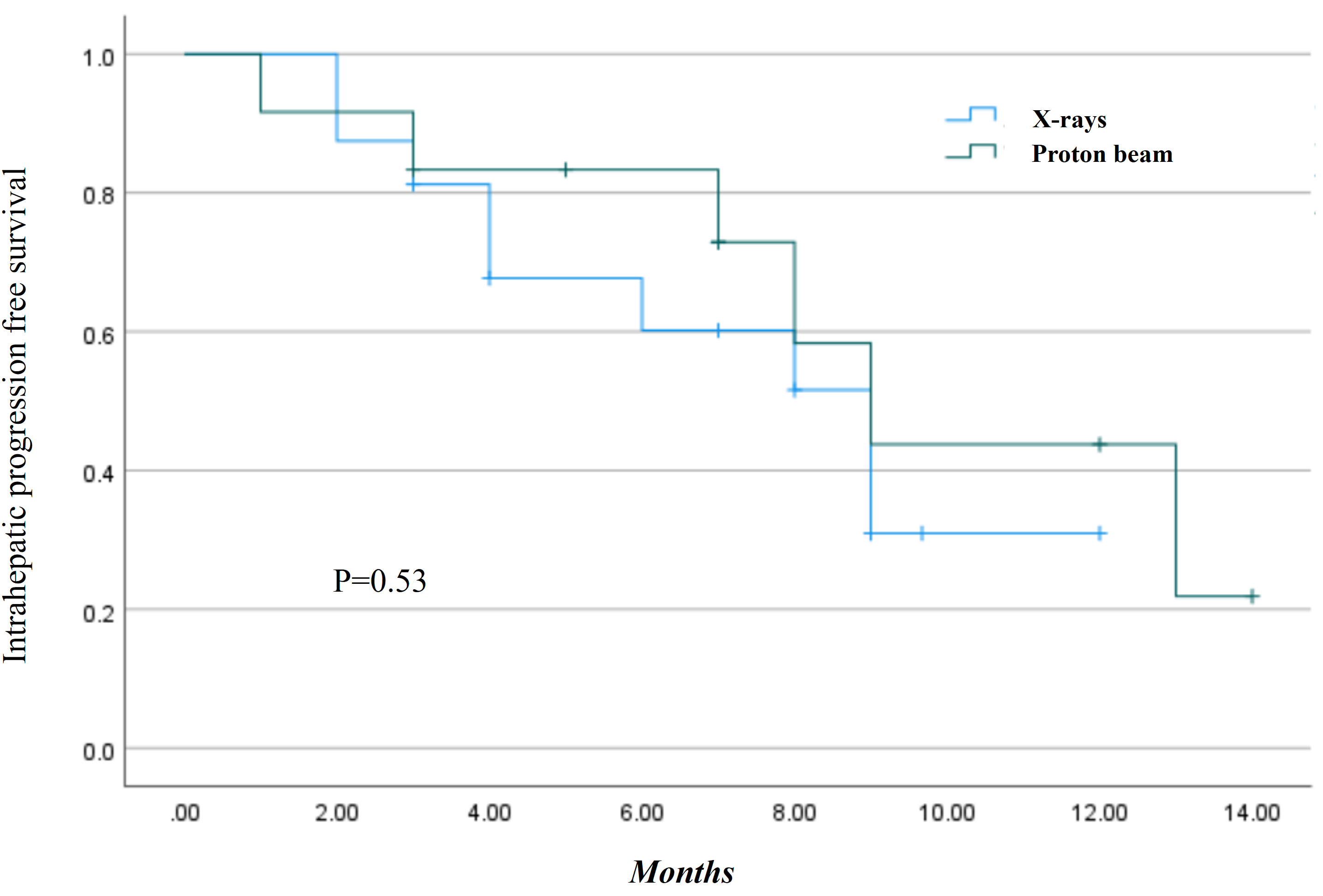


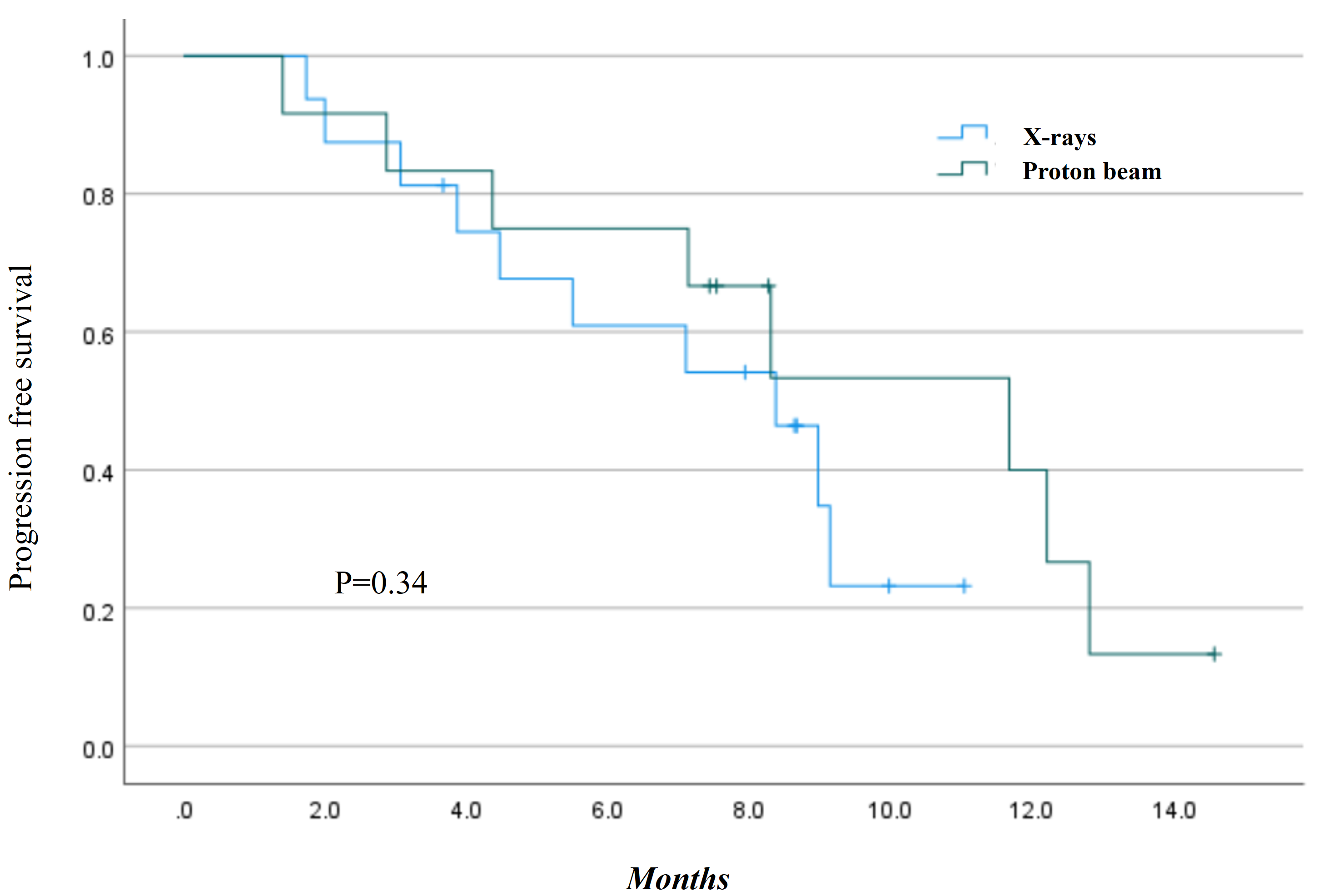


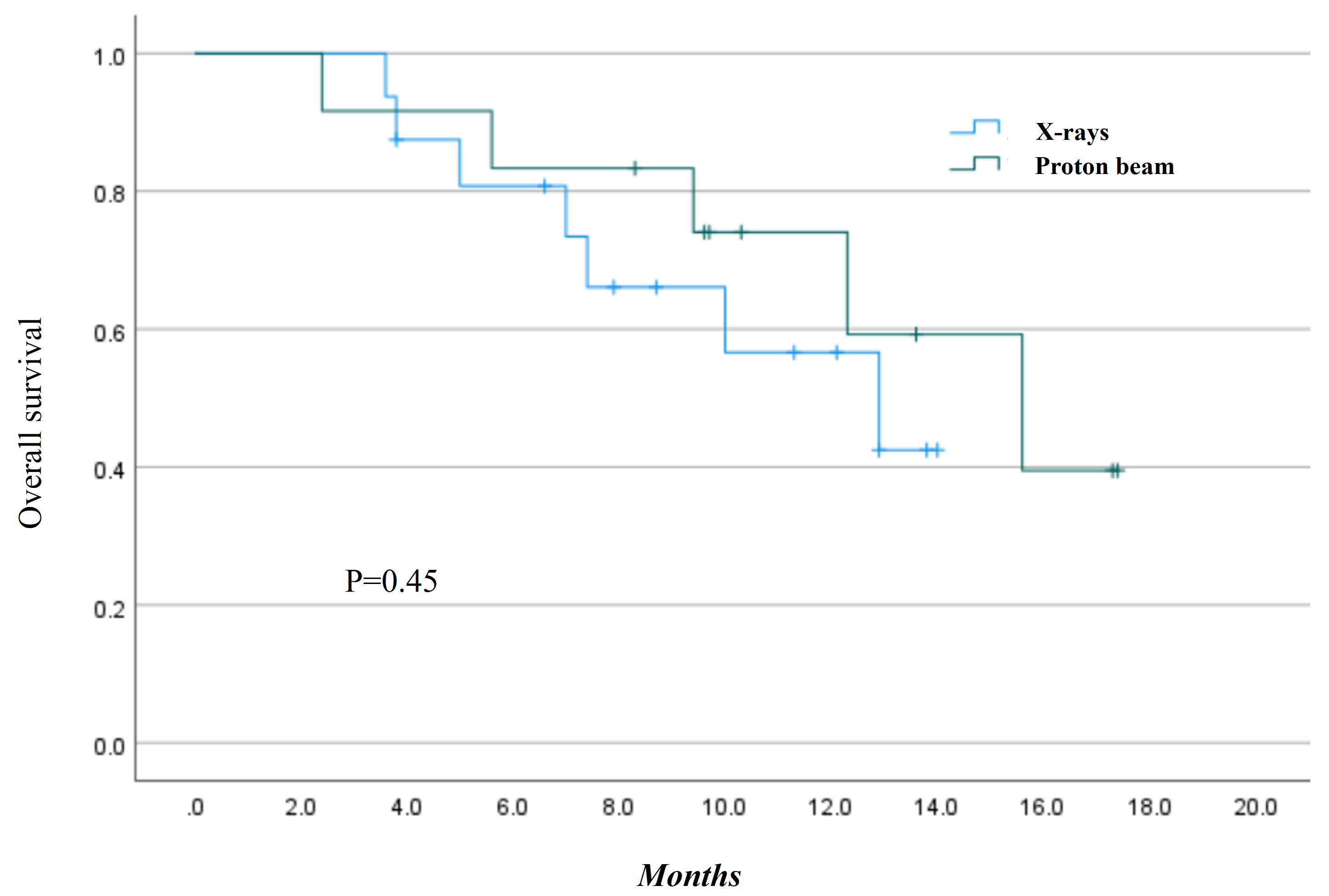


**Supplementary Figure 5 |** Kaplan-Meier estimated survival curves of intrahepatic-progression-free survival (IHPFS), progression-free survival (PFS), and overall survival (OS) according to the LRT targets. (TT, tumor thrombosis)


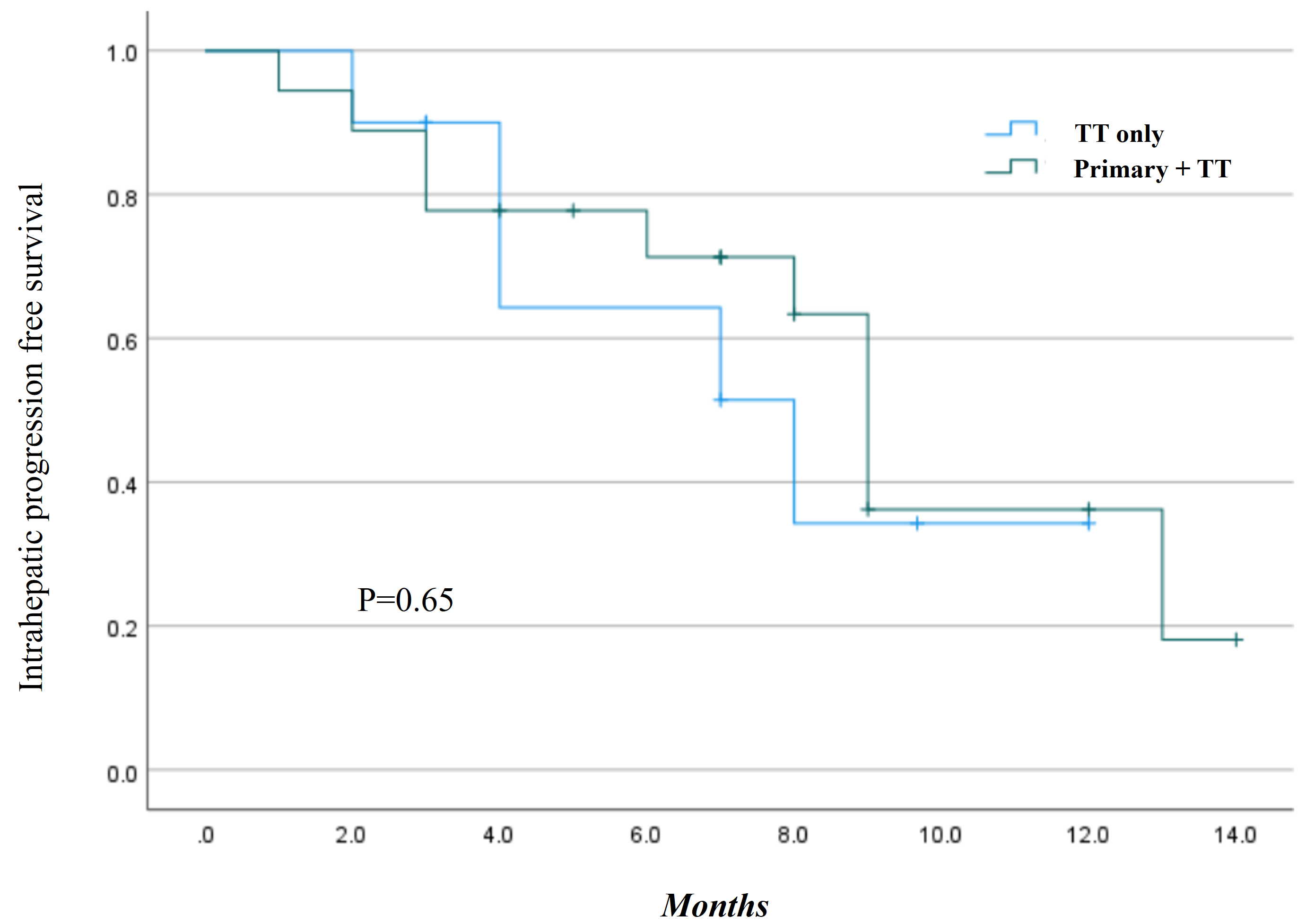


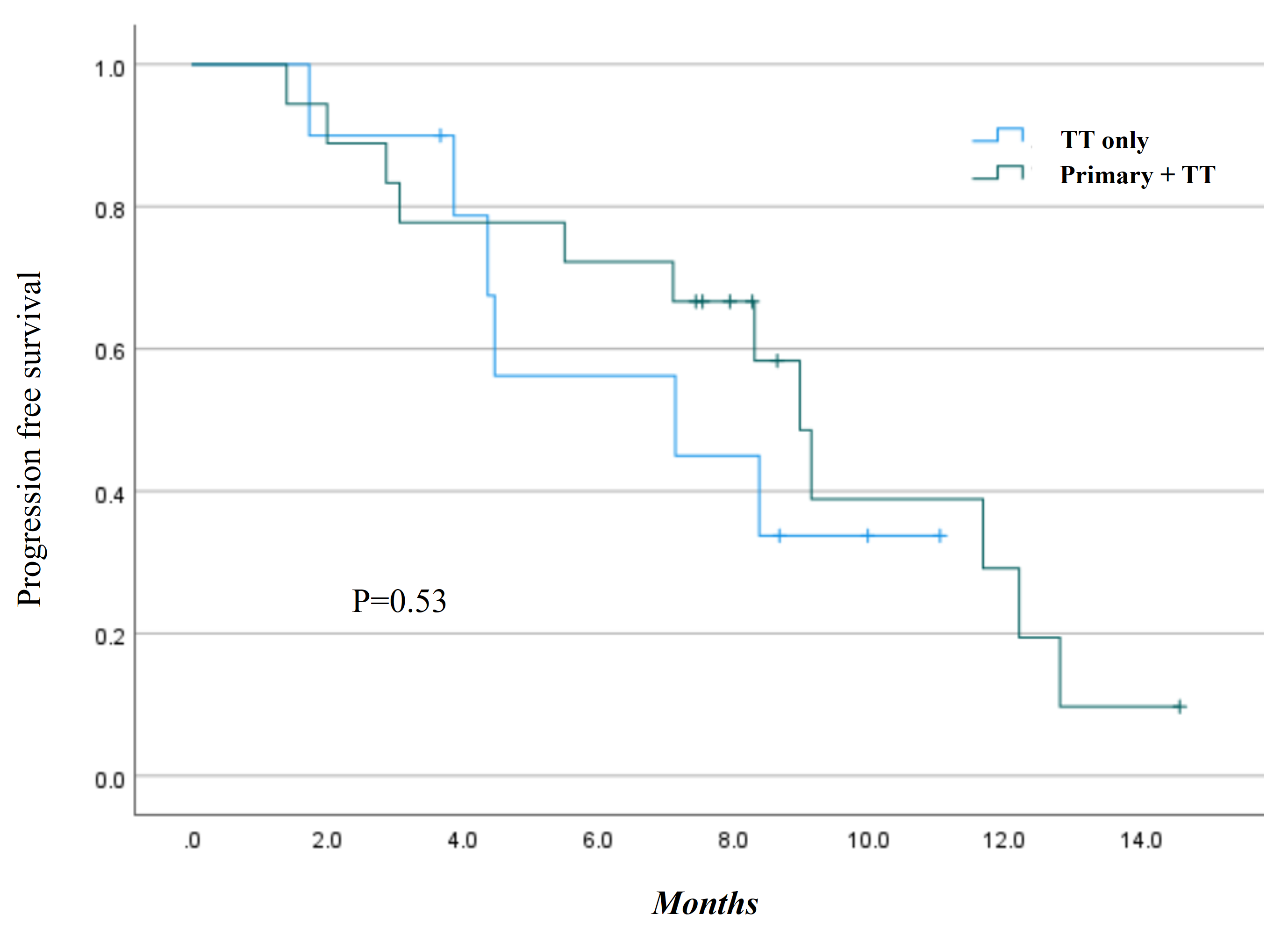


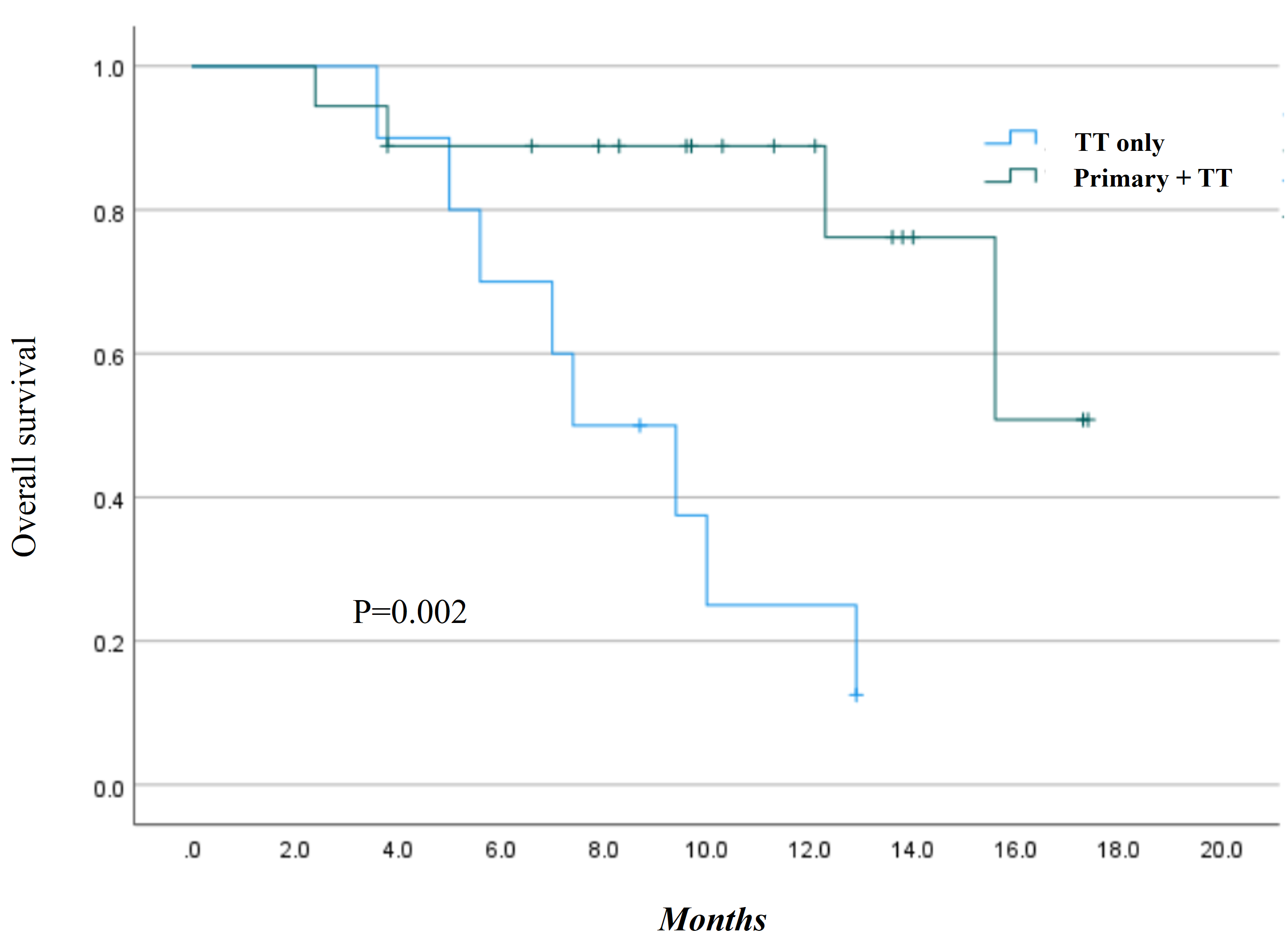

Supplement: Supplementary file 1 [file DataSheet_1.docx]
